# Socioeconomic status, individual behaviors and risk for Lymphomas: a Mendelian randomization study

**DOI:** 10.7150/jca.96413

**Published:** 2024-05-20

**Authors:** Zaixiang Tan, Ying Wang, Xing Xing, Ziyuan Shen, Wei Sang

**Affiliations:** 1 Research Center of Health Policy and Health Management, Xuzhou Medical University, Xuzhou 221004, Jiangsu, China.; 2 Department of Personnel, Suqian First Hospital, Suqian 223800, Jiangsu, China.; 3 Department of Epidemiology and Biostatistics, School of Public Health, Anhui Medical University, Hefei 230032, Anhui, China.; 4 Department of Hematology, The Affiliated Hospital of Xuzhou Medical University, Xuzhou 221006, Jiangsu, China.; 5 Blood Diseases Institute, Xuzhou Medical University, Xuzhou 221006, Jiangsu, China.; 6 Key Laboratory of Bone Marrow Stem Cell, Xuzhou 221006, Jiangsu, China.; 7 Cell Research and Translational Medicine Center, The Affiliated Hospital of Xuzhou Medical University, Xuzhou 221006, Jiangsu, China.

**Keywords:** Individual behavior, Lymphomas, Mendelian randomization, Socioeconomic status.

## Abstract

**Background**: The association of socioeconomic status and individual behavior (SES/IB) with human health is receiving increasing attention. However, the causal effects between SES/IB and lymphomas remain unclear.

**Methods:** A two-sample Mendelian randomization (MR) study was used to assess the causal effects of 25 SES/IB traits (dietary habits, physical activity, smoking/drinking behaviors, sleeping behaviors, leisure sedentary behaviors, risky behaviors, and reproductive behaviors) on six distinct types of lymphomas, including Hodgkin lymphoma (HL), follicular lymphoma (FL), diffuse large B-cell lymphoma (DLBCL), mature T/NK-cell lymphomas, marginal zone B-cell lymphoma (MZL), and mantle cell lymphoma (MCL). The inverse variance weighted (IVW) method was the primary approach used for the MR analysis. A series of sensitivity analyses were also conducted to ensure the robustness of the findings.

**Results**: Two-sample MR revealed six SES/IB traits causally associated with lymphomas, including relative fat intake, drive time, television watching time, computer use time, vigorous physical activity, and number of children ever born. After false discovery rate (FDR) correction, the causal associations between longer television watching time and DLBCL (*OR*: 4.048, 95% CI: 1.688 to 9.708, *P*_fdr_=0.009), and the number of children ever born with both FL (*OR*: 0.008, 95% CI: 1.412E-04 to 0.484, *P*_fdr_=0.021) and DLBCL (*OR*: 0.001, 95% CI:1.587E-05 to 0.081, *P*_fdr_=0.002) were identified.

**Conclusions**: These findings suggest that certain lifestyle and behavioral factors have a measurable impact on specific lymphoma types.

## Introduction

Lymphomas are highly heterogeneous hematological malignancies characterized by different etiology, symptoms, therapeutic approaches, and prognoses 1. Generally, lymphomas can be classified as Hodgkin's lymphoma (HL) and non-Hodgkin lymphoma (NHL), with HL making up about 10% of all lymphoma cases 2. The worldwide rise in lymphoma prevalence highlights the urgent need for earlier detection through improved diagnostics, identification of risk factors, and more effective treatments 3. Studies have demonstrated that genetic mutations, abnormal nutritional status (e.g., obesity), and virus infection (e.g., Epstein-Barr virus) are recognized risk factors for lymphomas 4-6.

The associations of socioeconomic status (SES, e.g., income, occupation, and education) and individual behaviors (IB, e.g., dietary habits, smoking, and sexual behavior) with human health have been identified in several diseases 7-9. Cai *et al.* 10 used Mendelian randomization (MR) to reveal causal associations between SES/IB and the onset of mental disorders. Moreover, a cross-sectional study suggested the association between SES/IB and the prevalence of metabolic syndrome and cardio-metabolic risk factors 11, highlighting the impact of SES/IB on health. Previous studies have suggested that SES/IB factors might influence the risk of lymphomas 12, 13. A meta-analysis has indicated a clear association between cigarette smoking and an elevated risk of lymphoma, particularly HL 14. Furthermore, a population-based case-control study 15 found a significant correlation between HL and employment in the manufacturing of rubber and plastic products. Additionally, it was observed that individuals working in metal processing faced a notably higher risk of developing diffuse large B-cell lymphoma (DLBCL). However, these observational studies are often confounded by numerous factors, making it challenging to infer causality. The causality underlying SES/IB and the risk of lymphomas remains unclear.

MR leverages genetic variations as instrumental variables to infer causal relationships between potentially modifiable factors and health outcomes 16. This study aimed to identify a total of 25 SES/IB-related traits associated with the risk of six distinct types of lymphomas based on MR analysis.

## Materials and Methods

### Study design

We employed a two-sample Mendelian randomization (MR) analysis to explore the causal effect between SES/IB (25 traits) and six unique lymphomas. MR leverages genetic variants as instrumental variables (IVs) to infer causality, capitalizing on inherent genetic predispositions. This approach rests on three fundamental assumptions 17: (1) the genetic variants are robustly associated with the exposure; (2) the variants are not linked with any confounders that could influence both the exposure and the outcome; and (3) the impact of the genetic variants on the outcome is mediated exclusively through the exposure. This genetic association study was conducted according to the Strengthening the Reporting of Observational Studies in Epidemiology using Mendelian Randomization (STROBE-MR) 18.

All studies incorporated into our analysis received approval from the respective institutional review boards, with participants providing informed consent.

### Genome-wide association study (GWAS) data for SES/IB-related factors

A total of 25 SES/IB phenotypes were included in this study (socioeconomic status, dietary composition, habitual physical activity, smoking, drinking, sleeping behaviors, leisure sedentary activities, risky behaviors, and reproductive habits). Genetic instruments for these phenotypes were obtained from publicly available summary-level data of 9 published GWAS 19-27. Detailed definitions and descriptions for each of the phenotypes were summarized in Supplementary Table 1.

### Data sources for lymphomas

This study encompassed six types of lymphomas. Data for Hodgkin lymphoma (HL; 846 cases and 324,650 controls), follicular lymphoma (FL; 1,181 cases and 324,650 controls), diffuse large B-cell lymphoma (DLBCL; 1,050 cases and 314,193 controls), mature T/NK-cell lymphomas (363 cases and 324,650 controls), marginal zone B-cell lymphoma (MZL; 202 cases and 314,193 controls), and mantle cell lymphoma (MCL; 210 cases and 314,193 controls) were publicly obtained from Release 10 of the FinnGen consortium 28, 29. There was little sample overlap between the exposures and outcomes populations. All participants are of European ancestry.

### Selection of IVs for MR analyses

The initial step involved selecting SNPs that reached genome-wide significance (*P*< 5×10^-8^). Subsequently, SNPs were further refined based on linkage disequilibrium (LD) thresholds, retaining those with an LD *r*² < 0.001 to ensure independence, where LD *r*² values were calculated using the 1000 Genomes Project reference panel. Additionally, to assess the strength of the IVs, we calculated the F-statistic for each IV, and if the F-statistic >10, it means that the possibility of weak IV bias is very small 30. For the relevance assumption, *R*^2^ was calculated to represent the proportion of variance in the exposure variable explained by the genetic variants.

### Statistical analysis

In this study, we employed a two-sample MR approach to reveal causal estimates of the effect of SES/IB on six unique lymphomas, including inverse variance weighting (IVW), MR-Egger, weighted median-based, weighted mode, and simple mode methods. The IVW can provide the most accurate estimates but is sensitive to horizontal pleiotropy and outliers, so IVW was used as the main method for this analysis with at least 2 genetic instruments. Conversely, the Wald ratio test was conducted for phenotypes with 1 genetic instrument 31. A *P* value <0.05 represented a statistically significant.

Sensitivity analyses were also conducted to ensure the robustness of the findings. The pleiotropy was assessed by using the MR‐Egger and weighted median methods. We also tested heterogeneity for IVW and MR‐Egger methods via Cochran's *Q* statistics and funnel plots 32. Leave-one-out analysis was used to identify SNPs with potential impacts and evaluate the reliability of the results. The adjusted *P*-values were also calculated using the false discovery rate (FDR) correction with the Benjamin-Hochberg method 33. The statistical significance of the causal feature was defined with FDR < 0.05.

For binary outcomes, an odds ratio (*OR*) and 95% confidence interval (*CI*) were applied to estimate the degree of a causal relationship. All analyses were performed in R software (version 4.2.2).

## Results

### The selected SNPs of exposures

After selecting genome-wide significant SNPs (*P*<5×10^-8^) and clumping at an LD threshold of r^2^ < 0.001, a total of 862 SNPs were identified as IVs for SES/IB-related factors. The F-statistics for these SES/IB-related SNPs were all greater than 10 (Supplementary Table 2, Datasets: 1-25).

### Causal effects of SES/IB-related phenotypes on lymphomas

**Figure 1:**
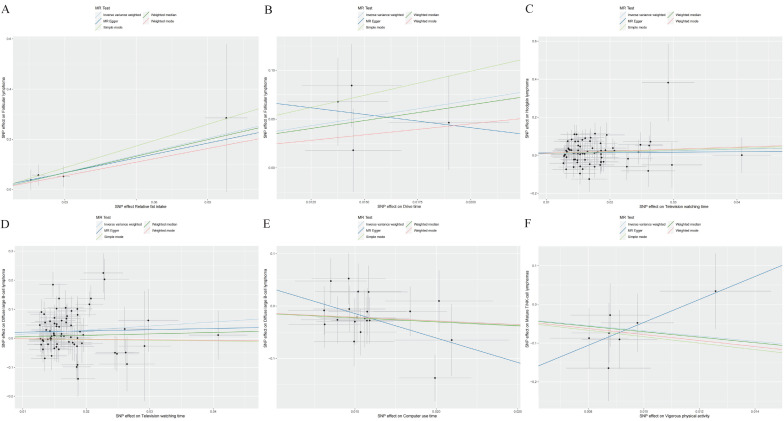
Causal associations of SES/IB-related factors with the risk of lymphomas. SNP: single nucleotide polymorphism. (A): Associations of Relative fat intake and follicular lymphoma (FL); (B): Associations of Drive time and FL; (C): Associations of Television watching time and Hodgkin lymphoma; (D): Associations of Television watching time and diffuse large B-cell lymphoma (DLBCL); (E): Associations of Computer use time and DLBCL; (F): Associations of Vigorous physical activity and mature T/NK-cell lymphomas.

A higher intake of fats and longer drive times were associated with increased risks of FL (*OR*: 9.642 to 31.799, *P*<0.05, Table 1 and Figure 1A, B) based on IVW results. Furthermore, extended periods of television watching were found to elevate the risk for both HL and DLBCL (*OR*: 2.187 to 4.048, *P*<0.05, Figure 1C, D).

Conversely, the time spent using a computer was inversely associated with the risk of DLBCL (*OR*=0.244, Figure 1E). Likewise, engaging in vigorous physical activities was linked to a significantly reduced risk of mature T/NK-cell lymphomas (*OR*=0.001, Figure 1F). The results indicated that having a higher number of children was associated with a decreased risk for both FL and DLBCL (*OR*: 0.001 to 0.008, *P*<0.05, Table 1).

Further examination applying FDR corrections confirmed that only the associations between television watching time and DLBCL (*P*_fdr_=0.009), and the number of children ever born with both FL (*P*_fdr_=0.021) and DLBCL (*P*_fdr_=0.002) remained significant.

### Sensitivity analyses

Sensitivity analysis results indicated that except for heterogeneity and pleiotropy between television watching time and DLBCL, there was no heterogeneity or pleiotropy between other SES/IB-related phenotypes and lymphomas (Supplementary Table 3). A positive association between longer television watching time and DLBCL was identified using MR-PRESSO after removing outliers (*OR* = 3.456, 95 % *CI*: 1.506-7.930, *P* = 0.005). Leave-one-out analysis confirmed the robustness of the MR results (Supplementary Figure 1).

## Discussion

In this study, we employed a two-sample MR analysis to identify SES/IB traits that have causal associations with lymphomas. Our findings indicated that long periods of television watching were linked to higher risks of both HL and DLBCL. Conversely, having more children was associated with a reduced risk of both FL and DLBCL. Additionally, engaging in vigorous physical activity appears to protect against mature T/NK-cell lymphomas. These results highlight the significant impact of SES/IB traits on lymphomas, suggesting potential approaches for prevention and intervention.

MR analysis suggested that extended periods of driving were genetically associated with an increased risk of FL, and long television watching time was associated with DLBCL risk. These associations may indicate a link between sedentary behavior (implicit in prolonged driving) and the risk of developing lymphomas. In line with our findings, a meta-analysis provides complementary evidence from a behavioral perspective. Specifically, it was found that individuals with a greater body mass index (BMI) during young adulthood faced an increased risk of FL (*OR* = 1.15) 34. This finding underscores the potential impact of lifestyle factors, such as sedentary behaviors, on FL risk. Given that extended driving periods can contribute to a sedentary lifestyle and potentially higher BMI, this suggests a multifaceted relationship between lifestyle behaviors and FL risk.

Ahmadi *et al.* 35 observed that engaging in vigorous activity for approximately 15-20 minutes weekly correlated with lower mortality, cardiovascular disease, and cancer incidence. Our MR analysis revealed that vigorous physical activity lowers the risk of mature T/NK-cell lymphomas, likely due to its anti-inflammatory effects. First, vigorous physical activities contribute to lowering visceral fat, which is a significant contributor to systemic inflammation 36. Second, vigorous activities enhance insulin signaling and glucose transport, resulting in improved insulin sensitivity. This is crucial because high insulin levels are associated with chronic inflammation 37. Moreover, vigorous exercise increases the production of anti-inflammatory agents, leading to a broad reduction in inflammation 38.

The current study revealed that having more children was associated with a reduced risk of both FL and DLBCL. This observation was consistent with findings from earlier studies, which demonstrated a decrease in NHL risk associated with a greater number of pregnancies among women who have given birth 39-42. This association may be attributed to a combination of hormonal, immunological, and lifestyle factors induced by pregnancy and parenthood. Increased exposure to reproductive hormones through multiple pregnancies and the use of external hormones may decrease the risk of NHL by influencing various aspects of the immune system 43, 44. This includes modulating cell-mediated immune (Th1) and antibody-mediated immune (Th2) responses, regulating the production of Th1 and Th2 cytokines, affecting the creation and longevity of B-cells, and impacting the apoptosis of immune cells 45. Furthermore, the maternal immune system is more effective in identifying and eliminating potential cancer cells during and after pregnancy. This may be due to the need to protect the mother and fetus from infection, inadvertently reducing the risk of cancer development. The adoption of healthier behaviors and lifestyle modifications that often accompany parenthood, especially with an increased number of children, could also explain this association. This may include changes in dietary habits, physical activity, stress management, and enhanced social support networks, which collectively can improve general health and possibly reduce the risk of cancer 46, 47. Nonetheless, the exact pathways through which these changes affect lymphomas are still not clear, and more studies are needed.

Moreover, the variable impact of similar SES/IB traits on different types of lymphoma may be related to the inherent biological differences among lymphoma subtypes, such as specific genetic mutations, molecular pathways, and immune responses. Certain mutations (e.g., BCL2, MYC) might make a subtype more aggressive or influence how it interacts with the hormonal or metabolic pathways of the body 48. These genetic distinctions could modulate the effect of environmental factors associated with SES/IB. Additionally, the immune system plays a critical role, and SES/IB can influence its effectiveness. Since different lymphoma subtypes may provoke and interact with the immune system differently, the impact of SES/IB could be manifested distinctively across these subtypes.

This study has several strengths. First, we assessed the causal effects between SES/IB traits and lymphoma risk through genetics for the first time. Second, we minimized observational biases such as confounding factors and reverse causality by employing MR analyses. However, several limitations of the current study should be noted. First, the limited sample size of lymphoma cases within the FinnGen cohort may compromise the reliability of the analysis outcomes, and the study population was restricted to individuals of European descent. Second, the lymphoma data sourced from the GWAS database lacked specific information on patient age and disease stage which could not conduct the detailed subgroup analysis. Additionally, the genetic instruments used to represent SES/IB might not capture the full range of these complex constructs, and the potential of residual confounding remains.

In conclusion, these results suggest that certain lifestyle and behavioral factors have a measurable impact on the risk of developing specific lymphoma types. Further research is needed to elucidate the mechanisms underlying these associations and to explore the potential for lifestyle modifications in lymphoma prevention.

## Supplementary Material

Supplementary figure and tables.

## Figures and Tables

**Table 1:** Two-sample Mendelian randomization analyses for the associations of SES/IB-related factors with the risk of lymphomas

Exposure-outcome	Methods	Number of SNPs	*OR*	95% *CI*	*P* value
Relative fat intake-**FL**	MR Egger	4	7.152	0.028 to 1812.71	0.558
	Weighted median	4	9.075	0.946 to 87.086	0.056
	**Inverse variance weighted**	4	9.642	1.294 to 71.835	**0.027**
	Simple mode	4	17.946	1.044 to 308.572	0.141
	Weighted mode	4	6.124	0.434 to 86.512	0.272
Drive time-**FL**	MR Egger	4	0.066	1.234E-11 to 3.568E+08	0.835
	Weighted median	4	25.424	0.713 to 907.039	0.076
	**Inverse variance weighted**	4	31.799	1.848 to 547.031	**0.017**
	Simple mode	4	141.942	1.328 to 15173.838	0.129
	Weighted mode	4	9.563	0.089 to 1026.669	0.414
Number of children ever born-**FL**	**Wald ratio**	1	0.008	1.412E-04 to 0.484	**0.021**
Television watching time-**HL**	MR Egger	72	1.237	0.034 to 45.277	0.908
	Weighted median	72	2.213	0.705 to 6.945	0.173
	**Inverse variance weighted**	72	2.187	1.023 to 4.674	**0.043**
	Simple mode	72	2.587	0.178 to 37.508	0.488
	Weighted mode	72	2.890	0.221 to 37.705	0.421
Television watching time-**DLBCL**	MR Egger	72	1.526	0.023 to 99.105	0.843
	Weighted median	72	1.644	0.586 to 4.61	0.345
	**Inverse variance weighted**	72	4.048	1.688 to 9.708	**0.002**
	Simple mode	72	0.794	0.052 to 12.103	0.868
	Weighted mode	72	0.838	0.068 to 10.271	0.891
Computer use time-**DLBCL**	MR Egger	20	0.000	0 to 2.627	0.093
	Weighted median	20	0.220	0.034 to 1.446	0.115
	**Inverse variance weighted**	20	0.244	0.065 to 0.915	**0.036**
	Simple mode	20	0.227	0.01 to 5.219	0.366
	Weighted mode	20	0.241	0.008 to 6.988	0.418
Number of children ever born-**DLBCL**	**Wald ratio**	1	0.001	1.587E-05 to 0.081	**0.002**
Vigorous physical activity-**Mature T/NK-cell lymphomas**	MR Egger	7	7.607E+12	1.977E-09 to 2.898E+34	0.295
	Weighted median	7	0.001	3.881E-07 to 1.909	0.073
	**Inverse variance weighted**	7	0.001	1.766E-06 to 0.683	**0.038**
	Simple mode	7	2.400E-04	9.006E-10 to 63.954	0.239
	Weighted mode	7	4.197E-04	1.103E-09 to 155.929	0.280

Note: SES/IB: socioeconomic status and individual behaviors; FL: follicular lymphoma; HL: Hodgkin lymphoma; DLBCL: diffuse large B-cell lymphoma; OR: odds ratio; CI: confidence interval.

## References

[1] Smith A, Crouch S, Lax S, Li J, Painter D, Howell D (2015). Lymphoma incidence, survival and prevalence 2004-2014: sub-type analyses from the UK's Haematological Malignancy Research Network. British journal of cancer.

[2] Mugnaini EN, Ghosh N (2016). Lymphoma. Primary Care: Clinics in Office Practice.

[3] Shen Z, Tan Z, Ge L, Wang Y, Xing X, Sang W (2024). The global burden of lymphoma: estimates from the Global Burden of Disease 2019 study. Public health.

[4] Xiong J, Cui BW, Wang N, Dai YT, Zhang H, Wang CF (2020). Genomic and Transcriptomic Characterization of Natural Killer T Cell Lymphoma. Cancer cell.

[5] Tse E, Zhao WL, Xiong J, Kwong YL (2022). How we treat NK/T-cell lymphomas. Journal of hematology & oncology.

[6] Castillo JJ, Ingham RR, Reagan JL, Furman M, Dalia S, Mitri J (2014). Obesity is associated with increased relative risk of diffuse large B-cell lymphoma: a meta-analysis of observational studies. Clinical Lymphoma Myeloma and Leukemia.

[7] Schultz WM, Kelli HM, Lisko JC, Varghese T, Shen J, Sandesara P (2018). Socioeconomic status and cardiovascular outcomes: challenges and interventions. Circulation.

[8] Yang Y, Wang S, Chen L, Luo M, Xue L, Cui D (2020). Socioeconomic status, social capital, health risk behaviors, and health-related quality of life among Chinese older adults. Health and quality of life outcomes.

[9] Huang G, Cai J, Li W, Zhong Y, Liao W, Wu P (2021). Causal relationship between educational attainment and the risk of rheumatoid arthritis: a Mendelian randomization study. BMC rheumatology.

[10] Cai J, Wei Z, Chen M, He L, Wang H, Li M (2022). Socioeconomic status, individual behaviors and risk for mental disorders: a Mendelian randomization study. European Psychiatry.

[11] Hao Z, Wang M, Zhu Q, Li J, Liu Z, Yuan L (2022). Association between socioeconomic status and prevalence of cardio-metabolic risk factors: a cross-sectional study on residents in North China. Frontiers in Cardiovascular Medicine.

[12] Odutola MK, Nnakelu E, Giles GG, van Leeuwen MT, Vajdic CM (2020). Lifestyle and risk of follicular lymphoma: a systematic review and meta-analysis of observational studies. Cancer Causes & Control.

[13] Davies GA, Strader C, Chibbar R, Papatheodorou S, Dmytriw AA (2020). The relationship between physical activity and lymphoma: a systematic review and meta analysis. BMC cancer.

[14] Sergentanis TN, Kanavidis P, Michelakos T, Petridou ET (2013). Cigarette smoking and risk of lymphoma in adults: a comprehensive meta-analysis on Hodgkin and non-Hodgkin disease. European Journal of Cancer Prevention.

[15] Mester B, Nieters A, Deeg E, Elsner G, Becker N, Seidler A (2006). Occupation and malignant lymphoma: a population based case control study in Germany. Occupational and environmental medicine.

[16] Evans DM, Davey Smith G (2015). Mendelian Randomization: New Applications in the Coming Age of Hypothesis-Free Causality. Annual review of genomics and human genetics.

[17] Scosyrev E (2013). Identification of causal effects using instrumental variables in randomized trials with stochastic compliance. Biometrical journal Biometrische Zeitschrift.

[18] Skrivankova VW, Richmond RC, Woolf BA, Yarmolinsky J, Davies NM, Swanson SA (2021). Strengthening the reporting of observational studies in epidemiology using Mendelian randomization: the STROBE-MR statement. Jama.

[19] Lee JJ, Wedow R, Okbay A, Kong E, Maghzian O, Zacher M (2018). Gene discovery and polygenic prediction from a genome-wide association study of educational attainment in 1.1 million individuals. Nat Genet.

[20] Hill WD, Davies NM, Ritchie SJ, Skene NG, Bryois J, Bell S (2019). Genome-wide analysis identifies molecular systems and 149 genetic loci associated with income. Nat Commun.

[21] Klimentidis YC, Raichlen DA, Bea J, Garcia DO, Wineinger NE, Mandarino LJ (2018). Genome-wide association study of habitual physical activity in over 377,000 UK Biobank participants identifies multiple variants including CADM2 and APOE. International journal of obesity (2005).

[22] Liu M, Jiang Y, Wedow R, Li Y, Brazel DM, Chen F (2019). Association studies of up to 1.2 million individuals yield new insights into the genetic etiology of tobacco and alcohol use. Nat Genet.

[23] Lane JM, Jones SE, Dashti HS, Wood AR, Aragam KG, van Hees VT (2019). Biological and clinical insights from genetics of insomnia symptoms. Nat Genet.

[24] Dashti HS, Jones SE, Wood AR, Lane JM, van Hees VT, Wang H (2019). Genome-wide association study identifies genetic loci for self-reported habitual sleep duration supported by accelerometer-derived estimates. Nat Commun.

[25] van de Vegte YJ, Said MA, Rienstra M, van der Harst P, Verweij N (2020). Genome-wide association studies and Mendelian randomization analyses for leisure sedentary behaviours. Nat Commun.

[26] Karlsson Linnér R, Biroli P, Kong E, Meddens SFW, Wedow R, Fontana MA (2019). Genome-wide association analyses of risk tolerance and risky behaviors in over 1 million individuals identify hundreds of loci and shared genetic influences. Nat Genet.

[27] Barban N, Jansen R, de Vlaming R, Vaez A, Mandemakers JJ, Tropf FC (2016). Genome-wide analysis identifies 12 loci influencing human reproductive behavior. Nat Genet.

[28] Leinonen JT, FinnGen, Pirinen M, Tukiainen T (2024). Disentangling the link between maternal influences on birth weight and disease risk in 36,211 genotyped mother-child pairs. Communications Biology.

[30] Pierce BL, Ahsan H, VanderWeele TJ (2011). Power and instrument strength requirements for Mendelian randomization studies using multiple genetic variants. International journal of epidemiology.

[31] Chen D, Zhang Y, Yidilisi A, Xu Y, Dong Q, Jiang J (2022). Causal associations between circulating adipokines and cardiovascular disease: a Mendelian randomization study. The Journal of Clinical Endocrinology & Metabolism.

[32] Xing X, Wang Y, Pan F, Cai G (2023). Osteoarthritis and risk of type 2 diabetes: A two-sample Mendelian randomization analysis. Journal of diabetes.

[33] Storey JD, Tibshirani R (2003). Statistical significance for genomewide studies. Proceedings of the National Academy of Sciences.

[34] Linet MS, Vajdic CM, Morton LM, de Roos AJ, Skibola CF, Boffetta P (2014). Medical history, lifestyle, family history, and occupational risk factors for follicular lymphoma: the InterLymph Non-Hodgkin Lymphoma Subtypes Project. Journal of the National Cancer Institute Monographs.

[35] Ahmadi MN, Clare PJ, Katzmarzyk PT, del Pozo Cruz B, Lee IM, Stamatakis E (2022). Vigorous physical activity, incident heart disease, and cancer: how little is enough?. European Heart Journal.

[36] Flynn MG, McFarlin BK (2006). Toll-like receptor 4: link to the anti-inflammatory effects of exercise?. Exercise and sport sciences reviews.

[37] Mathur N, Pedersen BK (2008). Exercise as a mean to control low-grade systemic inflammation. Mediators of inflammation. 2008.

[38] Petersen AMW, Pedersen BK (2005). The anti-inflammatory effect of exercise. Journal of applied physiology.

[39] Lee JS, Bracci PM, Holly EA (2008). Non-Hodgkin lymphoma in women: reproductive factors and exogenous hormone use. American Journal of Epidemiology.

[40] Zhang Y, Holford TR, Leaderer B, Boyle P, Zahm SH, Zhang B (2004). Menstrual and reproductive factors and risk of non-Hodgkin's lymphoma among Connecticut women. American journal of epidemiology.

[41] Costas L, Casabonne D, Benavente Y, Becker N, Boffetta P, Brennan P (2012). Reproductive factors and lymphoid neoplasms in Europe: findings from the EpiLymph case-control study. Cancer Causes & Control.

[42] Kane E, Roman E, Becker N, Bernstein L, Boffetta P, Bracci P (2012). Menstrual and reproductive factors, and hormonal contraception use: associations with non-Hodgkin lymphoma in a pooled analysis of InterLymph case-control studies. Annals of oncology.

[43] Costas L, de Sanjosé S, Infante-Rivard C (2014). Reproductive factors and non-Hodgkin lymphoma: a systematic review. Critical Reviews in Oncology/Hematology.

[44] Shah MR, Brandt JS, David KA, Evens AM (2020). Lymphoma occurring during pregnancy: current diagnostic and therapeutic approaches. Current oncology reports.

[45] Lang TJ (2004). Estrogen as an immunomodulator. Clinical immunology.

[46] Abu-Raya B, Michalski C, Sadarangani M, Lavoie PM (2020). Maternal immunological adaptation during normal pregnancy. Frontiers in immunology.

[47] Jørgensen N, Persson G, Hviid TVF (2019). The tolerogenic function of regulatory T cells in pregnancy and cancer. Frontiers in immunology.

[48] Schuetz J, Johnson N, Morin R, Scott D, Tan K, Ben-Nierah S (2012). BCL2 mutations in diffuse large B-cell lymphoma. Leukemia.

