# Supplementary figures and images for: Socioeconomic status, individual behaviors and risk for Lymphomas: a Mendelian randomization study
Source: J Cancer. 2024 May 20;15(12):3760–5. doi: 10.7150/jca.96413 (PMC11190779; doi:10.7150/jca.96413)

A

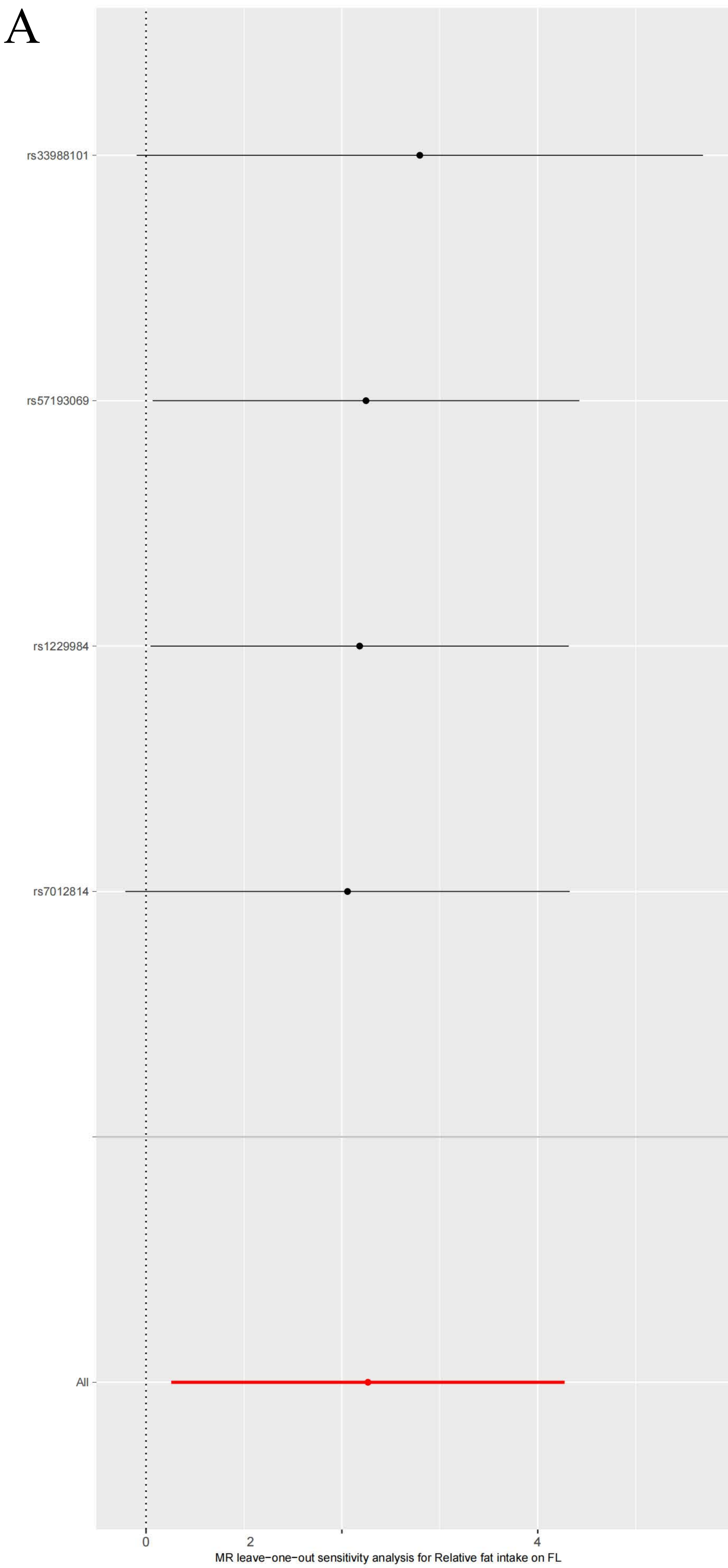

B

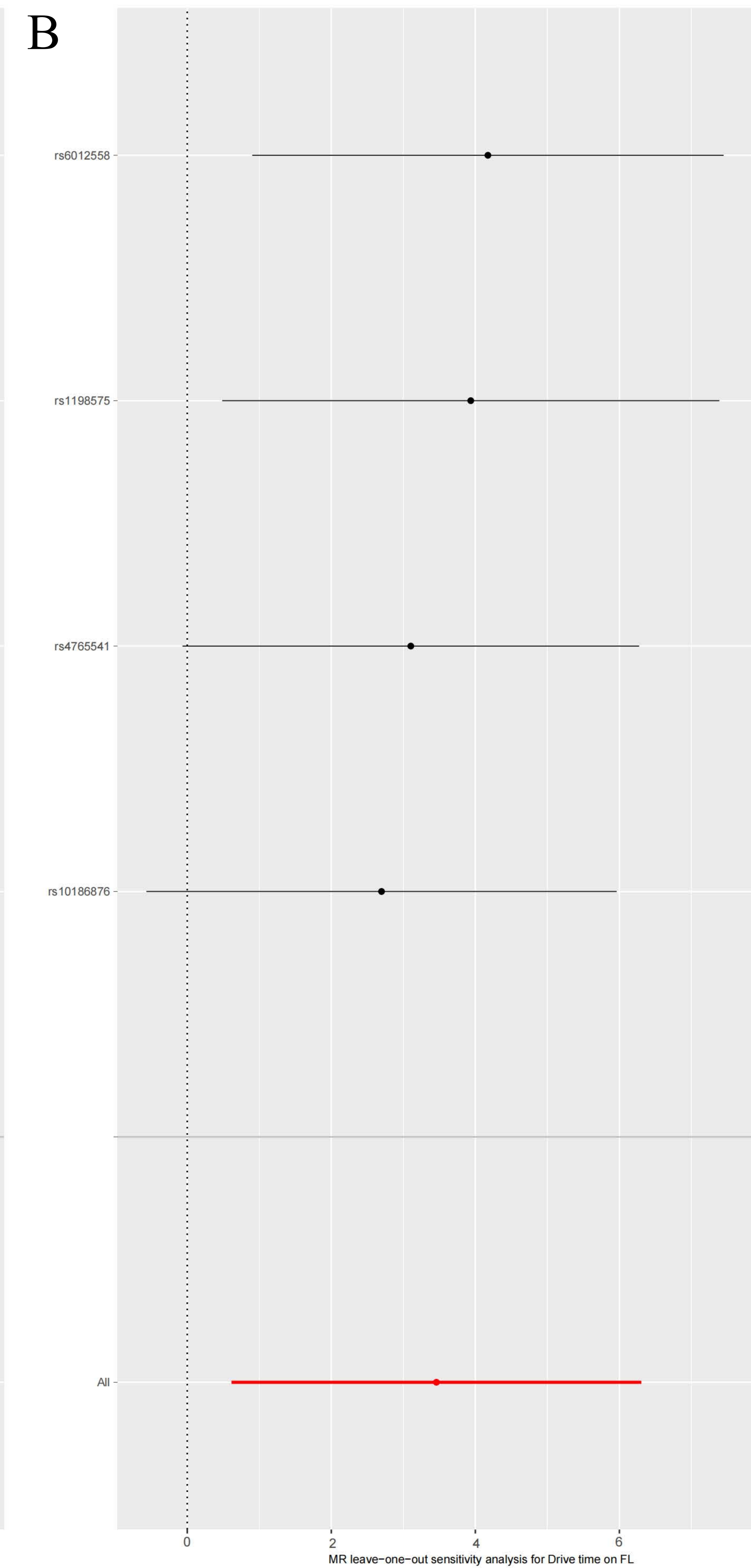

C

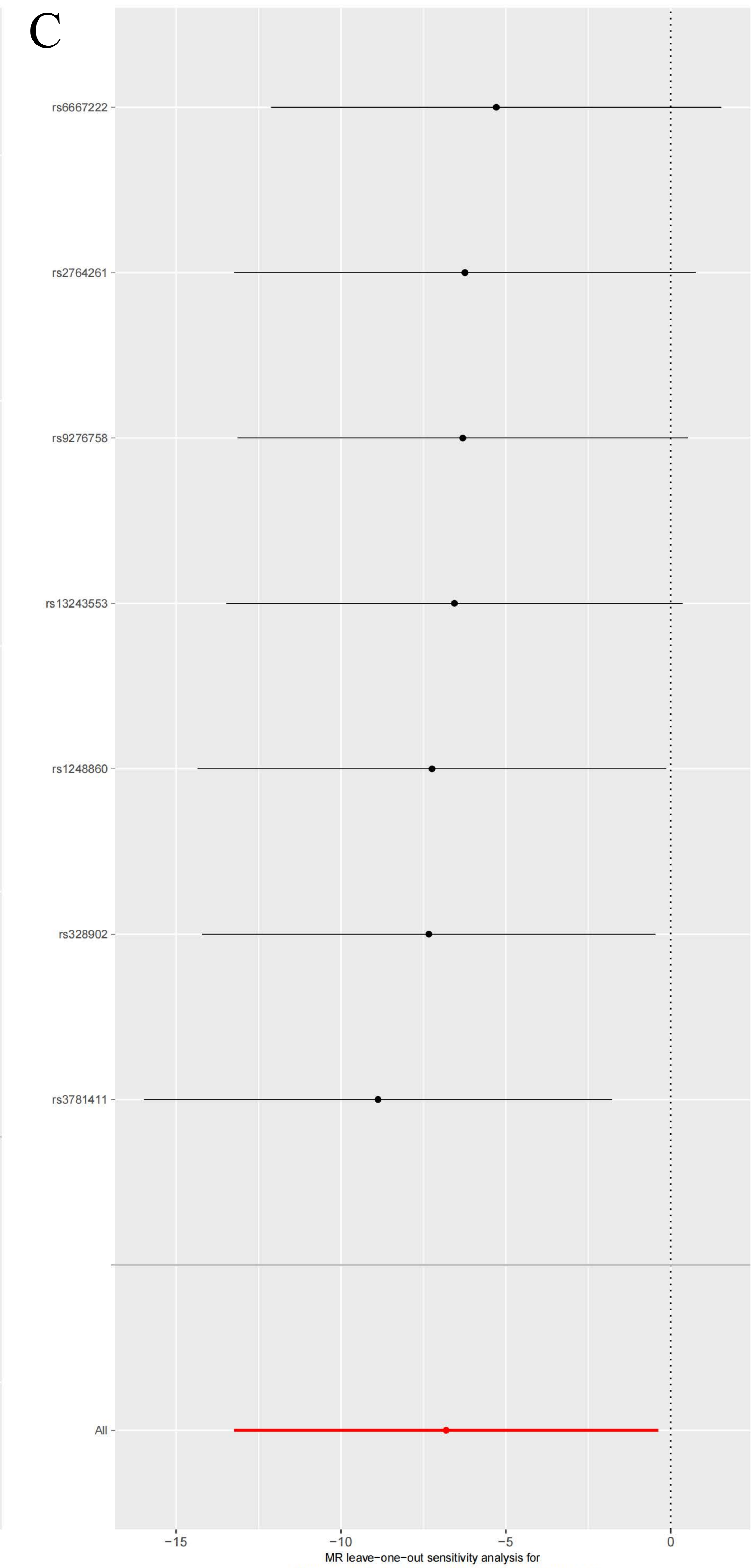

D

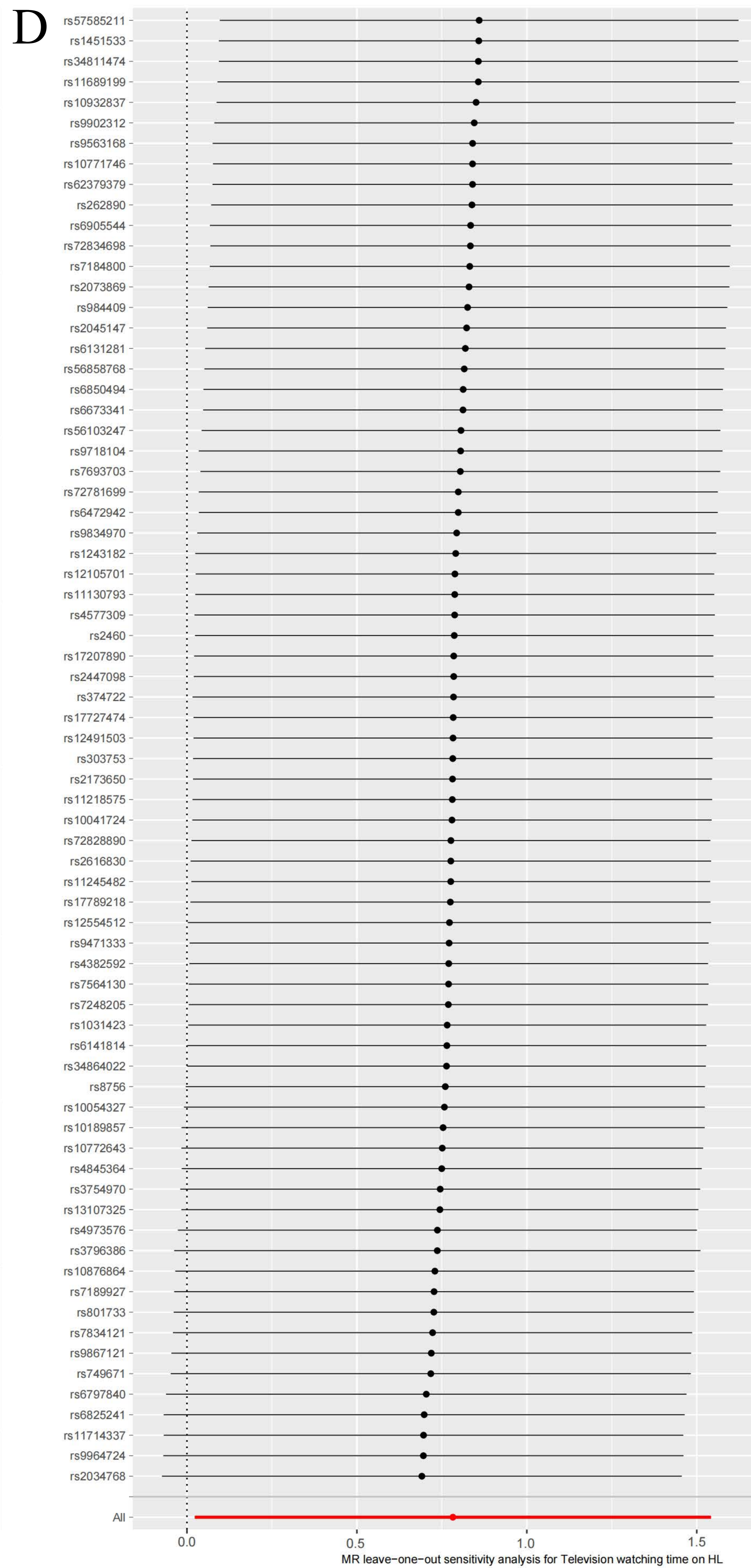

E

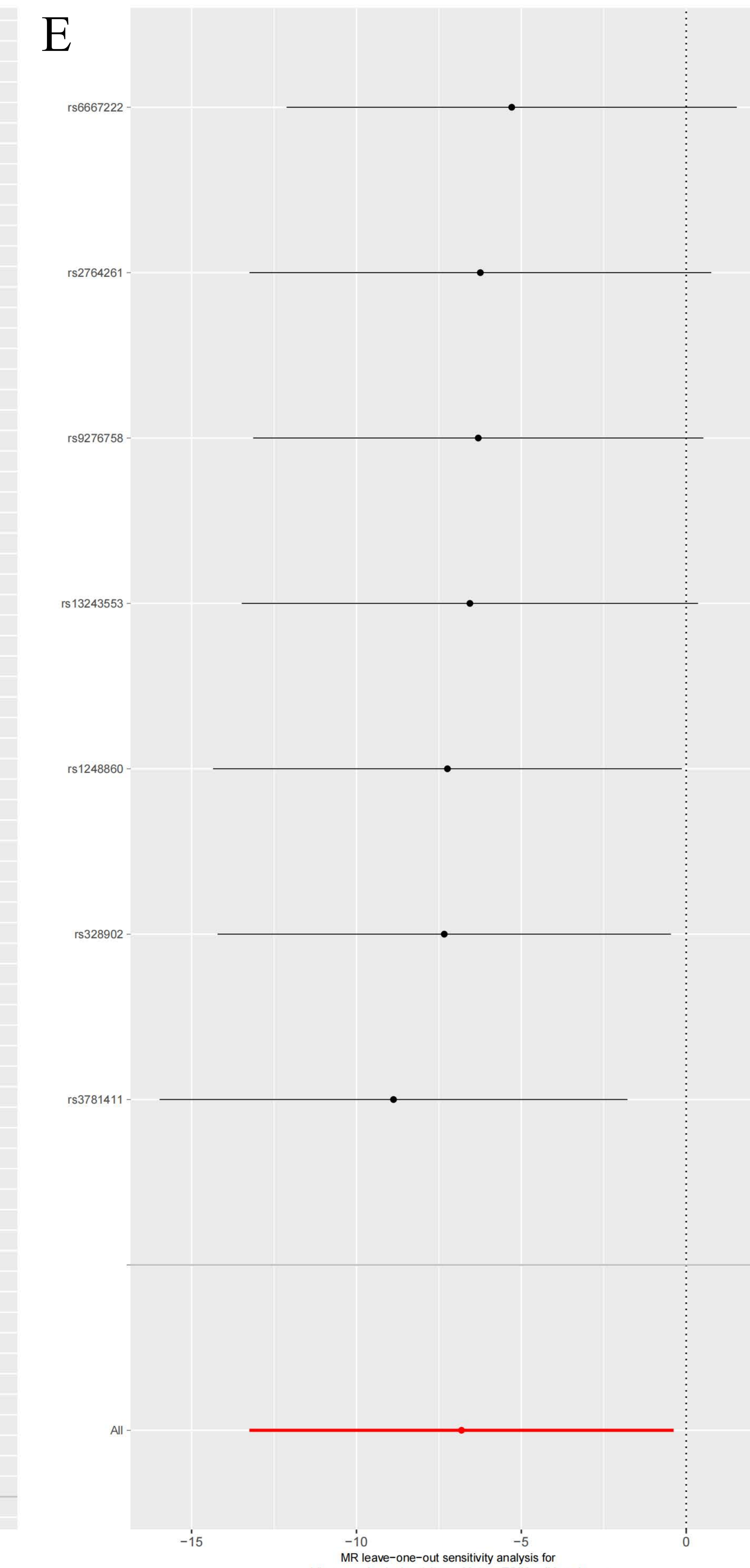

Supplement: Supplementary file 1 — Supplementary figure and tables. [file jcav15p3760s1.zip › Supplementary files/Supplementary Figure 1.pdf]
